# A speedup technique for (*l, d*)-motif finding algorithms

**DOI:** 10.1186/1756-0500-4-54

**Published:** 2011-03-08

**Authors:** Sanguthevar Rajasekaran, Hieu Dinh

**Affiliations:** 1 Department of CSE, University of Connecticut, Storrs, CT 06269, USA

## Abstract

**Background:**

The discovery of patterns in DNA, RNA, and protein sequences has led to the solution of many vital biological problems. For instance, the identification of patterns in nucleic acid sequences has resulted in the determination of open reading frames, identification of promoter elements of genes, identification of intron/exon splicing sites, identification of SH RNAs, location of RNA degradation signals, identification of alternative splicing sites, etc. In protein sequences, patterns have proven to be extremely helpful in domain identification, location of protease cleavage sites, identification of signal peptides, protein interactions, determination of protein degradation elements, identification of protein trafficking elements, etc. Motifs are important patterns that are helpful in finding transcriptional regulatory elements, transcription factor binding sites, functional genomics, drug design, etc. As a result, numerous papers have been written to solve the motif search problem.

**Results:**

Three versions of the motif search problem have been proposed in the literature: *Simple Motif Search *(*SMS*), (*l, d*)*-motif search (or Planted Motif Search (PMS))*, and *Edit-distance-based Motif Search (EMS)*. In this paper we focus on PMS. Two kinds of algorithms can be found in the literature for solving the PMS problem: *exact *and *approximate*. An exact algorithm identifies the motifs always and an approximate algorithm may fail to identify some or all of the motifs. The exact version of PMS problem has been shown to be NP-hard. Exact algorithms proposed in the literature for PMS take time that is exponential in some of the underlying parameters. In this paper we propose a generic technique that can be used to speedup PMS algorithms.

**Conclusions:**

We present a speedup technique that can be used on any PMS algorithm. We have tested our speedup technique on a number of algorithms. These experimental results show that our speedup technique is indeed very effective. The implementation of algorithms is freely available on the web at http://www.engr.uconn.edu/rajasek/PMS4.zip

## Background

Pattern search in biological sequences has numerous applications and hence a large amount of research has been done to identify patterns. Motifs are fundamental functional elements in proteins vital for understanding gene function, human disease, and may serve as therapeutic drug targets. Three versions of the motif search problem have been identified by researchers: *Simple Motif Search (SMS)*, *Planted Motif Search (PMS) *- *also known as *(*l, d*)-*motif search*, and *Edit-distance-based Motif Search (EMS) *(see e.g., [1]).

PMS problem takes as input *n *sequences of length *m *each and two integers *l *and *d*. The problem is to identify a string *M *of length *l *such that *M *occurs in each of the *n *sequences with a Hamming distance of at most *d*. For example, if the input sequences are GCGCGAT, CACGTGA, and CGGTGCC; *l *= 3 and *d *= 1, then GGT is a motif of interest.

EMS is the same as PMS, except that edit distance is used instead of the Hamming distance. SMS takes as input *n *sequences and an integer *l*. The problem is to identify all the patterns of length *l *(with up to *l*/2 wild card characters), together with a count of how many times each pattern occurs.

Two kinds of algorithms can be found in the literature for the solution of PMS. The first kind of algorithms identify all the motifs always. This kind of algorithms are called *exact algorithms*. The second kind of algorithms may not always identify the motif(s). Numerous algorithms of each kind can be found in the literature. The exact version of the PMS problem is known to be NP-complete.

Some example approximate algorithms are due to [2-5], and [6]. These algorithms employ local search techniques such as Gibbs sampling, expectation optimization, etc. The WINNOWER algorithm in [5] is based on finding cliques in a graph. The PROJECTION algorithm of [3] employs random projections. Approximate algorithms tend to be very fast but there is no guarantee that we will get all the motifs of interest. Other examples of approximate algorithms include: MULTIPROFILER [7], PatternBranching [8], CONSENSUS [9], GibbsDNA [4], MEME [2], and ProfileBranching [8].

Several exact algorithms are also known for solving the PMS problem: [10-16], and [17]. PMS algorithms are typically tested on random benchmark data generated as follows: Twenty sequences each of length 600 are generated randomly from the alphabet of interest. The motif *M *is also generated randomly and planted in each of the input sequences within a Hamming distance of *d*. The motif instances are also generated randomly. Certain instances of the (*l*, *d*)-motif problem have been identified to be *challenging*. An instance is challenging if the expected number of (*l*, *d*)-motifs that occur by random chance (in addition to the planted one) is one or more. For example, the following instances are challenging: (9, 2), (11, 3), (13, 4), (15, 5), (17, 6), (19, 7), etc. The performance of PMS algorithms are customarily shown only for challenging instances.

The exact algorithm MITRA of [8] can solve the challenging instance (15, 4). It cannot solve (15, 5) or any larger instances. On these instances it takes either too much time or too much space. Three exact algorithms PMS1, PMS2, and PMS3 have been given in [18]. These algorithms are faster than MITRA. Other exact algorithms are: Voting of [19], RISOTTO of [20], and PMSprune of [21].

In this paper we present a speedup technique that can be used for any PMS algorithm. Before presenting details of our technique, we provide a brief summary of the following algorithms: PMS1, Voting, RISOTTO, and PMSprune. We have employed these algorithms to demonstrate the efficacy of our technique.

## Methods

### A Summary of Known Algorithms

#### PMS0 and PMS1

PMS0 and PMS1 are two exact algorithms given in [18]. PMS0 works as follows. Let *S*_1_, *S*_2_, ..., *S_n _*be the given input sequences and let *m *be the length of each input sequence. For any two *l*-mers *u *and *v *let *H.D*.(*u*,*v*) stand for the Hamming distance between *u *and *v*. Let *u *be any *l*-mer. We define the *d*-neighborhood of *u *(denoted as *D_u_*) as the set of *l*-mers that are at a distance of ≤ *d *from *u*. In other words, *D_u _*is nothing but {*v*: *H*.*D*.(*u*, *v*) ≤ *d*}. Let *C *be the collection of *l*-mers in *S*_1_. Note that *C *has (m - *l *+ 1) *l*-mers. Let *C' *= ∪_*u*∈*C*_*Du*. Note that . For each element *v *of *C*' check if it is a valid (*l*, *d*)-motif or not. Given an *l*-mer *v*, we can check if it is a valid (*l*, *d*)-motif or not in *O*(*mnl*) time. Thus the run time of PMS0 is .

PMS1 is an exact algorithm that works as follows. For each input sequence *S_i _*(1 ≤ *i *≤ *n*) it generates a list of d-neighbors. In particular, for each *l*-mer *u *in *S_i_*, it generates *D_u_*. It merges all such *D_u_*'s and eliminates duplicates to get *L_i_*. The output (*l*, *d*)-motifs will be . More details follow.

**Algorithm **PMS1

1. Generate all possible *l*-mers from out of each of the *n *input sequences. Let *C_i _*be the collection of *l*-mers from out of *S_i _*for 1 ≤ *i *≤ *n*.

2. For all 1 ≤ *i *≤ *n *do: ;

3. Sort all the *l*-mers in every , 1≤ *i *≤ *n*, in lexicographic order, and eliminate duplicates in every . An integer sorting algorithm can be used (see e.g., [22]). Let *L_i _*be the resultant sorted list corresponding to .

4. Merge all the *L_i_*'s (1 ≤ *i *≤ *n*) and output the generated (in step 2) *l*-mers that occur in all the *L_i_*'s.

The following theorem results.

**Theorem 0.1 ***PMS1 runs in time **where w is the word length of the computer*.

#### Voting

The Voting algorithm proposed in [19] is very similar to PMS1. In this algorithm also, the potential motifs considered are the *d*-neighbors of each *l*-mer in the input sequences. In particular, they employ a hash table *V *of *d*-neighbors. Each such *d*-neighbor collects votes. Let *v *be a *d*-neighbor of some *l*-mer in the input. Then, *v *will receive a vote from the input sequence *i *(for 1 ≤ *i *≤ *n*) if *v *is a *d*-neighbor of some *l*-mer in the input sequence *S_i_*. They ensure that v will not get multiple votes from any sequence using another hash table *R*.

The algorithm builds both *V *and *R *by processing each *l*-mer *u *in each input sequence, generating the *d*-neighborhood of *u*, and hashing the *d*-neighbors into *V *and *R*. After processing all the input *l*-mers in this fashion, the algorithm outputs all those *d*-neighbors that receive a vote of *n*.

Clearly, the asymptotic run time of this algorithm is  assuming that *l *is a constant. If *l *is not of constant size, there could be a linear dependence on *l *as well in the run time.

#### RISOTTO

An exact algorithm for PMS has been given by [23] that has a run time of *O*(*n*^2^*ml^d^*|Σ|*^d^*). This algorithm uses *O*(*n*^2^*m*/*w*) space where *w *is the word length of the computer. This algorithm constructs a suffix tree on the input sequences in *O*(*nm*) time using *O*(*nm*) space. Some preprocessing is done on the suffix tree that takes *O*(*n*^2^*m*/*w*) time and *O*(*n*^2^*m*/*w*) space. If *u *is any *l*-mer in any input sequence, then *u *has *O(l^d^*(|Σ| - 1)*^d^*) possible *d*-neighbors. Any of these neighbors could potentially be a motif of interest. Since there are *O*(*nm*) *l*-mers in the input, the number of such neighbors is *O *(*nml^d^*(|Σ| - 1)*^d^*. For each such neighbor *v *the algorithm of [23] walks through the suffix tree to check if *v *is a valid motif (i.e., it has a *d*-neighbor in each input sequence). This walking step is referred to as 'spelling'. The spelling operation takes a total of *O*(*n*^2^*ml^d^*(|Σ| - 1)*^d^*) time using an additional *O*(*nm*) space.

An improved version of the above algorithm, called RISOTTO, has been given by [20]. The algorithm of [23] makes use of a trie called the motif tree. The root of this tree corresponds to the empty string. The algorithm grows this string one symbol at a time and for each such string checks if it is a valid motif (i.e., the string is of the right length and it occurs in every sequence within a Hamming distance of *d*). A key observation that [20] make use of in their algorithm is the following. Let *q *be a string that occurs in all the input sequences (up to a Hamming distance of *d*). Let its *maximum extensibility *be *MaxExt*(*q*).

*MaxExt*(*q*) refers to the maximum number of symbols that can be appended to *q *so that the resultant string will occur in all the input sequences (within a Hamming distance of *d*). If *q*' is another string such that *q *is a suffix of *q*', then *MaxExt*(*q*') cannot be more than *MaxExt*(*q*). As a result, if *MaxExt*(*q*') + |*q*'| <*l*, then we don't have to consider augmenting *q*' further. In other words, we can prune the subtree rooted at *q*' in the motif tree [20]. show that the average performance of RISOTTO is better than that of [23].

#### PMSprune

PMSprune follows the same strategy as PMS0: for every *l*-mer *y *in *S*_1 _it generates the set of neighbors of *z *and for each one of them checks whether this is a valid (*l, d*)-motif or not. However it improves the performance of PMS0 in a significant way. Salient features of this algorithm are:

1. It generates the neighborhood of every *l*-mer *u *in a branch and bound manner. In this method, these *l*-mers will correspond to nodes in a tree of height at most *d*. The root (which is at level zero) of this tree will be *u*. At level *i *of this tree they generate *l*-mers that are at a distance of *i *from *u *(for 1 ≤ *i *≤ *d*).

2. Let *S *= {*S*_1_, *S*_2_, ..., *S_n_*} be the input set of sequences. If *x *is any *l*-mer, the distance between *x *and any input sequence *S_i _*is denoted as *d*(*x, S_i_*) and is defined as min {*H*.*D*.(*x, y*)|*y *is an *l *- mer in *S_i_*}.

The distance between *x *and *S *is denoted as  and is defined as .

Let *u *be any *l*-mer in *S*_1 _and let *x *be any *l*-mer in the *d*-neighborhood of *u *(i.e., *D_u_*). PMSprune checks if *x *is a valid (*l, d*)-motif or not by computing . If  ≤ *d *then *x *is output - it is a valid motif. More importantly, if *x *is at level *h *in the tree, PMSprune uses the value of *d*(*x*, *S*) and *h *to prune the descendants of *x*. In particular, if  >(2*d *- *h*), then the subtree rooted at *x *is pruned - none of the *l*-mers in this subtree could be a valid (*l*, *d*)-motif.

3. It dynamically prunes the *l*-mers in *S_i _*for *i *= 2, ..., *n *that are considered for the calculation of .

4. It calculates the value of  in an incremental way taking into account the way the neighborhood is generated.

The worst case run time of PMSprune is  and it uses *O*(*nm*^2^) space. Even though its worst case run time is worse than that of PMS0, PMSprune has a better expected run time and it does better in practice [21].

### Our Speedup Technique

#### Summary

We refer to our speedup technique as PMS4 and it can be used in conjunction with any PMS algorithm. The idea of PMS4 is also based on PMS0. We can think of PMS0 as consisting of two stages. In the first stage, we generate all the *l*-mers of *S*_1 _and for each such *l*-mer generate its *d*-neighborhood. All of these *d*-neighborhoods are then merged to get the list *L*_1 _of *l*-mers. In the second stage, for each *l*-mer *v *in *L*_1_, check if *v *is a valid (*l, d*)-motif or not.

In other words, in stage 1 we come up with a list of candidate motifs. In the second stage, for each candidate motif we check if it is a valid motif or not. For each candidate motif it takes *O*(*mnl*) time to check if it is a valid motif or not. This is rather a large amount of time. PMS0 does not perform well since there are a large number of candidate motifs and for each candidate motif it takes a long time to check its validity. We can speedup its performance if we can reduce the number of candidate motifs (and/or if we can speedup the validity checking for each candidate motif).

PMS4 reduces the number of candidate motifs by first running the PMS algorithm on a small number of input sequences. It then verifies the validity of each candidate. Let  be the PMS algorithm under concern. A pseudocode for PMS4 follows.

**Algorithm **PMS4

1. Run the algorithm  on *k *input sequences (where *k *<*n*). An optimal value of *k *can be determined empirically. We could pick the *k *sequences in a number of ways. For example, they could be the first *k *sequences, random *k *sequences, and so on. Let *C *be the collection of (*l, d*)-motifs found in these *k *sequences.

2. **for **each *l*-mer *v *in C **do**

Check if *v *is a valid motif in *O*(*mnl*) time. If so, output *v*.

#### A probabilistic analysis

The problem of planted motif search is complicated by the fact that, for a given value of *l*, if the value of *d *is large enough, then the expected number of motifs that occur by random chance could be enormous. For instance, when *n *= 20, *m *= 600, *l *= 9, *d *= 2, the expected number of spurious motifs (that occur in each input sequence at a hamming distance of *d*) is 1.6. On the other hand for *n *= 20, *m *= 600, *l *= 10, *d *= 2, the expected number of spurious motifs is only 6.1 × 10^-8^. A probabilistic analysis to this effect can be conducted as follows (as shown in [3]).

Let *S_k _*be any input sequence 1 ≤ *k *≤ *n *and let *u *be any *l*-mer. Probability that *u *occurs in *S_k _*at a Hamming distance of *d *starting from a specific position is  Thus, probability that *u *occurs in *S_k _*starting from at least one of the positions in *S_k _*is 1 - (1 - *p*)^*m*-*l*+1^. Here it is assumed that the occurrence of *u *is independent of the starting position (which is clearly not true). Buhler and Tompa argue that this assumption nearly holds in practice [3]. This means that the expected number of *l*-mers that occur in each of the input sequences (at a hamming distance of *d*) is 4*^l ^*[1 - (1 - *p*)^*m-l*+1^]*^n^*.

A slightly different valid analysis has been presented in [24]. Let *S_k _*be any input sequence 1 ≤ *k *≤ *n *and let *u *be any *l*-mer. Call the positions *special positions*. Probability that *u *occurs in *S_k _*at a hamming distance of *d *starting from a specific special position is . Thus, probability that *u *occurs in *S_k _*starting from at least one of the special positions is 1 - (1 - *p*)*^m' ^*where . As a result, probability that *u *occurs somewhere in *S_k_*is at least 1 - (1 - *p*)^*m*'^. This means that the expected number of *l*-mers that occur in each of the input sequences (at a hamming distance of *d*) is ≥ 4*^l ^*[1 - (1 - *p*)^*m*'^]*^n^*.

Table 1 shows the expected number of motifs for different values of *l*, *d*, and *k*. In this table E.N.M. stands for the expected number of motifs. E.N.M. values in this table have been computed using the first (inaccurate) analysis.

**Table 1:** Expected number of motifs for various values of *l*, *d*, and *k*

E.N.M. stands for the expected number of motifs.											
***l***	***d***	***k***	**E.N.M.**	***l***	***d***	***k***	**E.N.M.**	***l***	***d***	***k***	**E.N.M.**

9	2	8	1,383	11	3	8	9,297	13	4	8	42,337

9	2	9	718	11	3	9	4,331	13	4	9	16,855

9	2	10	373	11	3	10	2,018	13	4	10	6,710

9	2	11	194	11	3	11	940	13	4	11	2,672

9	2	12	101	11	3	12	438	13	4	12	1,064

15	5	8	145,959	17	6	8	407,602	19	7	8	968,241

15	5	9	47,962	17	6	9	107,681	19	7	9	201,530

15	5	10	15,761	17	6	10	28,448	19	7	10	41,947

15	5	11	5,179	17	6	11	7,516	19	7	11	8,731

15	5	12	1,702	17	6	12	1,986	19	7	12	1,818

The run times of many of the known PMS algorithms are linearly dependent on the number of input sequences. Examples include PMS0, PMS1, RISOTTO, Voting, and PMSprune. Any reduction in the number of input sequences will result in a corresponding reduction in the run time. If the number of resultant motifs is small then the overall run time will be reduced. We have to strike a balance between the time it takes for the first stage and the second stage. A good starting point for the value of *k *is ⌈*n*/2⌉. We could then work around this value to optimize the time.

## Results and Discussion

We have tested the performance of PMS1, PMSprune, and RISOTTO for various values of (*l*, *d*), and *k*. The improvements in performance are shown next. We have not included Voting in this comparison since the Voting program takes a very long time when we decrease the number of sequences. When we decrease the number of sequences the number of motifs increases. The Voting program sorts these motifs and outputs them. We suspect that the sorting program used could be the reason for the long run times. However, since the asymptotic run time of Voting is linear in the number of sequences, PMS4 is expected to result in a speedup of Voting similar to that in PMS1 and RISOTTO. All the times reported in this section are averages over 10 random instances (fixing the values of *k*, *l*, and *d*). Each instance is a benchmark set of 20 random sequences of length 600 as mentioned in the Background Section

### The case of (9, 2)

**Figure 1:**
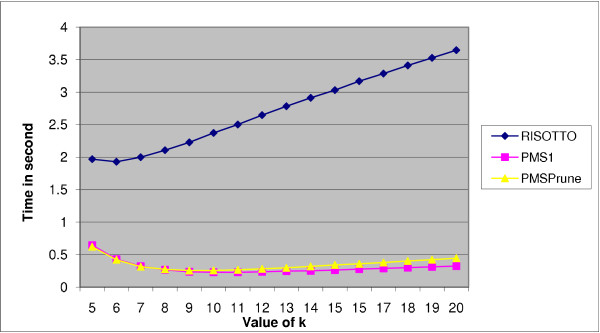
**Performance of PMS1, PMSprune, and RISOTTO for *l *= 9, *d *= 2, and various values of *k***. The *x*-axis corresponds to the values of *k *and the *y*-axis corresponds to the run times in seconds.

Results for the case of *l *= 9 and *d *= 2 are shown in Table 2. In this table, we display the time taken by each algorithm for *k *= 20 (denoted as *T*_20_) in column 2. Note that these algorithms, by default, work with 20 sequences. The best times found using PMS4 (denoted as *T_b_*) are shown in column 3. The ratio *T*_20_/*T_b _*(called the speedup) is shown in column 4. It is clear from this table that each of these algorithms benefits from PMS4. In particular, RISOTTO benefits the most with a speedup of close to 2. Figure 1 shows the performance of these algorithms for various values of *k *starting from 5.

**Table 2:** The best speedups of PMS4 for *l *= 9, *d *= 2

Algorithm	Time for *k *= 20 (*T*_20_) in seconds	Best time using PMS4 (*T* _b_) in seconds	Speedup = *T* _20 _=*T_b_*
PMS1	0.3234	0.2279	1.42

PMSprune	0.4436	0.2545	1.74

RISOTTO	3.647	1.9282	1.89

### The case of (11, 3)

**Figure 2:**
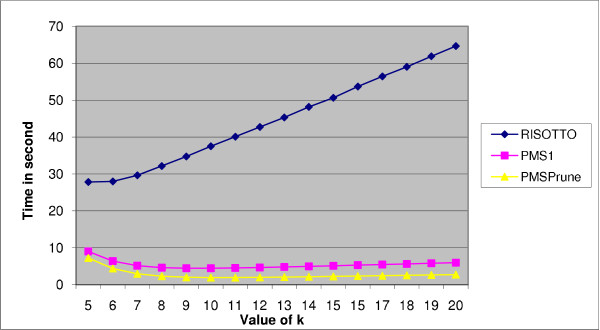
**Performance of PMS1, PMSprune, and RISOTTO for *l *= 11, *d *= 3, and various values of *k***. The *x*-axis corresponds to the values of *k *and the *y*-axis corresponds to the run times in seconds.

Results for the case of *l *= 11 and *d *= 3 are shown in Table 3. For this case also RISOTTO benefits the most with a speedup of more than 2. The speedup for PMSprune has decreased and that for PMS1 has also decreased but not by the same amount as PMSprune. Figure 2 shows the performance of these three algorithms for different values of *k*.

**Table 3:** The best speedups of PMS4 for *l *= 11, *d *= 3

Algorithm	Time for *k *= 20 (*T*_20_) in seconds	Best time using PMS4 (*T_b_*) in seconds	Speedup = *T* _20 _=*T_b_*
PMS1	5.9749	4.4469	1.34

PMSprune	2.7298	1.9218	1.42

RISOTTO	64.6362	27.8341	2.32

### The case of (13, 4)

**Figure 3:**
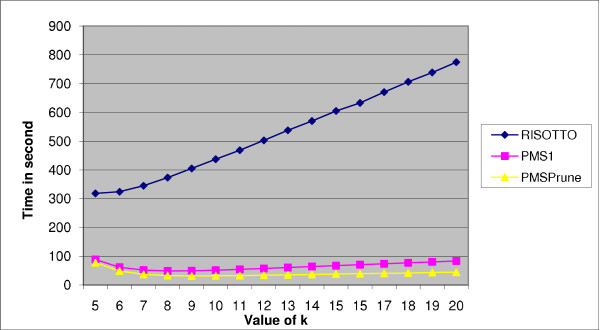
**Performance of PMS1, PMSprune, and RISOTTO for *l *= 13, *d *= 4, and various values of *k***. The *x*-axis corresponds to the values of *k *and the *y*-axis corresponds to the run times in seconds.

Table 4 summarizes the performance of PMS1, PMSprune and RISOTTO. The speedup for RISOTTO has increased. In general, the performance of RISOTTO increases with an increasing value of *l*. The performance of PMS1 also increases with an increasing value of *l*. However, the performance of PMSprune seems to be stable. Figure 3 displays the performance of all the three algorithms.

**Table 4:** The best speedups of PMS4 for *l *= 13, *d *= 4

Algorithm	Time for *k *= 20 (*T*_20_) in seconds	Best time using PMS4 (*T_b_*) in seconds	Speedup = *T* _20 _=*T_b_*
PMS1	83.7	49.2	1.70

PMSprune	44.6	31.6	1.41

RISOTTO	774.3	318.5	2.43

### The case of (15, 5)

**Figure 4:**
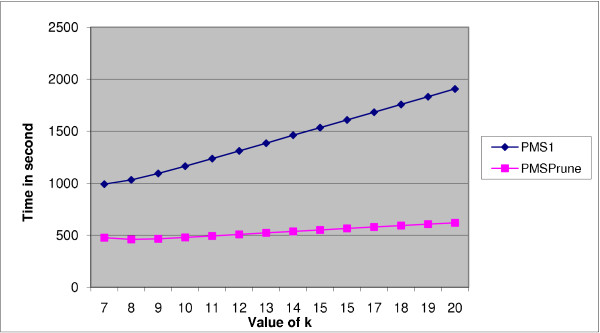
**Performance of PMS1 and PMSprune for *l *= 15, *d *= 5, and various values of *k***. The *x*-axis corresponds to the values of *k *and the *y*-axis corresponds to the run times in seconds.

In Table 5 and Figure 4, we show the results for PMS1 and PMSprune. RISOTTO takes too much time for this case and hence we have not included it in this comparison. As has been observed before, the speedup of PMS1 increases with an increasing value of *l *and the speedup for PMSprune stays nearly the same (at around 1.4).

**Table 5:** The best speedups of PMS4 for *l *= 15, *d *= 5

Algorithm	Time for *k *= 20 (*T*_20_) in seconds	Best time using PMS4 (*T_b_*) in seconds	Speedup = *T* _20 _=*T_b_*
PMS1	1906.7	990.7	1.92

PMSprune	619.2	460.1	1.35

Based on the experimental results, we suggest that the best value of *k *should be chosen around 0.4*n *to have maximum speedup.

## Conclusion

In this paper we have presented a speedup technique that can be used on any PMS algorithm. We have tested our speedup technique on a number of algorithms. These experimental results show that our speedup technique is indeed very effective.

## Competing interests

The authors declare that they have no competing interests.

## Authors' contributions

SR and HD designed and analyzed the algorithms. HD implemented the algorithms and carried out the empirical experiments. SR and HD analyzed the empirical results.

Both SR and HD read and approved this paper.
